# Determination of Activation Overpotential during the Nucleation of Hcp-Cobalt Nanowires Synthesized by Potentio-Static Electrochemical Reduction

**DOI:** 10.3390/ma11122355

**Published:** 2018-11-22

**Authors:** Ryusei Saeki, Takeshi Ohgai

**Affiliations:** 1 Graduate School of Engineering, Nagasaki University, Bunkyo-machi 1-14, Nagasaki 852-8521, Japan; bb52318101@ms.nagasaki-u.ac.jp; 2 Faculty of Engineering, Nagasaki University, Bunkyo-machi 1-14, Nagasaki 852-8521, Japan

**Keywords:** nanowire, cobalt, electrodeposition, overpotential, nucleation, magnetization

## Abstract

The crystal growth process and ferromagnetic properties of electrodeposited cobalt nanowires were investigated by controlling the bath temperature and cathodic overpotential. The cathodic overpotential during electrodeposition of cobalt nanowire arrays, Δ*E*^cath^, was theoretically estimated by the difference between the cathode potential, *E*^cath^, and the equilibrium potential, *E*^eq^, calculated by the Nernst equation. On the other hand, the activation overpotential, Δ*E*^act^, was experimentally determined by the Arrhenius plot on the growth rate of cobalt nanowire arrays, *R*_g_, versus (vs.) reciprocal temperature, 1/*T*. The ferromagnetic cobalt nanowire arrays with a diameter of circa (ca.) 25 nm had the preferred crystal orientation of (100) and the aspect ratio reached up to ca. 1800. The average crystal grain size, *Ds*, of (100) peaks was estimated by X-ray diffraction patterns and was increased by decreasing the cathodic overpotential for cobalt electrodeposition by shifting the cathode potential in the noble direction. Axial magnetization performance was observed in the cobalt nanowire arrays. With increasing *Ds*, coercivity of the film increased and reached up to ca. 1.88 kOe.

## 1. Introduction

The standard electrode potential of metallic cobalt (Co/Co^2+^ −0.28 V vs. normal hydrogen electrode (NHE)) is nobler than that of metallic iron (Fe/Fe^2+^ −0.44 V vs. NHE). Hence, cobalt-based magnetic materials, compared with iron-based, have excellent corrosion resistance when a magnet is used under a corrosive environment [1,2]. Cobalt alloy nanowires with a large aspect ratio show excellent magnetic performance, including large coercivity, large squareness and high Curie temperature [3,4,5]. Several fabrication processes of metallic alloy nanowire arrays have so far been reported by researchers. The template-based approach, using a thin film with numerous nanochannels such as ion-track etched polycarbonate templates and anodized aluminum oxide (AAO) templates [6,7,8,9,10,11,12], is a highly recommended process. The AAO nanochannels with numerous through-holes can realize straight nanowires with large aspect ratios.

Some researchers have reported the ferromagnetism of cobalt alloy nanowires, which were electrodeposited from an aqueous solution into the nanochannels of templates [13,14,15,16]. Pirota et al. reported that face centered cubic/ hexagonal close packed (fcc/hcp) bi-crystalline multilayer Co nanowire arrays were obtained by an electroplating method [17]. They have synthesized the dual-phase (fcc and hcp) Co nanowires (diameter, *D*, of 25–29 nm; length, *L*, of 1.13–2.25 µm; and aspect ratio, *L*/*D*, of 45–78) with a coercivity, *H*_c_, of up to ca. 1.0 kOe and squareness, *M*_r_/*M*_s_, of ca. 0.38. Vivas et al. investigated the effects of length on the coercivity of Co nanowire arrays that were fabricated by an electrodeposition process [18]. They have characterized the Co nanowires (diameter, *D*, of 35 nm; length, *L*, of 0.3–40 µm; and aspect ratio, *L*/*D*, of 9–1143) with a coercivity, *H*_c_, of up to ca. 1.3 kOe. Kaur et al. reported that structurally tailored cobalt nanowires were able to be electrochemically grown in the nanopores of commercially available AAO templates with a potentio-static electrochemical growth technique [19]. They obtained the structurally tailored Co nanowires (average diameter, *D*, of 20 nm; average length, *L*, of 1.6 µm; and average aspect ratio, *L*/*D*, of ca. 80) with a coercivity, *H*_c_, of 0.45 kOe and squareness, *M*_r_/*M*_s_, of ca. 0.3. Shaterabadi et al. also investigated the effect of solution pH on the ferromagnetism of cobalt nanowires that were electrochemically grown in the nanopores of an AAO layer on metallic aluminum films using an alternating current (AC) process [20]. They synthesized the dual-phase (fcc and hcp) Co nanowires (*D*: 35 nm, *L*: ranging from 1 µm to 5 µm, and *L*/*D*: ranging from 30 to 150) with an *H*_c_ of 1.45 kOe and *M*_r_/*M*_s_ of 0.89 (as-deposited). Srivastav et al. also studied the effects of solution pH on the ferromagnetism of cobalt nanowires, which were fabricated using a potentio-static deposition technique [21]. They discovered that the *H*_c_ and *M*_r_/*M*_s_ of the Co alloy nanowires (*D*: 70 nm, *L*: 10 µm, and *L*/*D*: ca. 150) were 0.84 kOe and 0.26, respectively (at room temperature).

The surface magnetic flux density of magnetic materials depends on the volume of the ferromagnetic phase. On the other hand, *H*_c_ of a ferromagnet decreases with the increase of average crystal grain size, which results in the formation of a multi-magnetic-domain structure. Therefore, if nanowires with a single-magnetic-domain structure are used as a permanent magnet with substantial volume, an industrial maker will require nanowires with a large aspect ratio. However, so far, the aspect ratio of cobalt nanowires with a coercivity of more than 2.5 kOe has not reached 1000. Recently, our research group reported that cobalt nanowires with a large aspect ratio (*L*/*D*: 1800) can be electrochemically grown from a bath containing CoCl_2_ and using AAO templates (*D*: ca. 25 nm) [22]. It is well known that acid halides in an aqueous solution accelerate the corrosion reaction of metallic materials and that this will cause harmful effects on the production lines. Hence, in this study, we tried to use non-halide type aqueous solutions containing sulfuric acid for the electrodeposition of cobalt nanowire arrays. We used AAO templates with aspect ratios of more than 1000.

## 2. Materials and Methods 

Pure aluminum rods (99%) with a diameter of 10 mm were anodically polished in an ethanol solution containing perchloric acid to smooth the surface of the cross-section. The polished aluminum rod was anodized in an aqueous solution containing 0.3 mol L^−1^ sulfuric acid by applying a constant cell voltage of 20 V for 24 h to form an AAO nanochannel layer (ca. 45 µm thickness) on the cross-section. During the anodization, the solution temperature was kept at 2 °C using an incubator with a cooling function. It is well known that highly ordered nanochannel structures can be made by the two-step anodization process [23]. Pirota et al. reported that Co nanowire arrays, which were electrodeposited into two-step anodized AAO nanochannels, exhibited a coercivity, *H*_c_, of up to ca. 1.0 kOe and squareness, *M*_r_/*M*_s_, of ca. 0.38 [17]. In our previous work we demonstrated that Co nanowire arrays that were electrodeposited into one-step anodized AAO nanochannels, showed a coercivity, *H*_c_ of up to ca. 2.4 kOe and squareness, *M*_r_/*M*_s_ of ca. 1.0 [16]. Hence, the size and crystallinity of a nanowire, the diameter, length and crystal orientation, are significant parameters in the ordering state of nanochannels. Therefore, in this study, a single step anodization process was employed to increase the experimental efficiency of making samples with a large aspect ratio. After the anodization, the nanochannel layer was separated from the aluminum rod by anodic etching of the interface between the AAO layer and the aluminum rod in an ethanol solution containing perchloric acid. The volume ratio between ethanol (99.5 wt.%) and perchloric acid (60 wt.%) was adjusted to 1:1. During the anodic etching, cell voltage was kept at 35 V for 30 s at room temperature. On one side of the AAO nanochannel templates, a thin gold layer was sputter-deposited to make a conductive layer as a cathode. For the electrodeposition of cobalt nanowire arrays, an aqueous solution containing 0.5 mol L^−1^ cobalt sulfate heptahydrate and 0.4 mol L^−1^ boric acid was synthesized using ultra-pure water (resistivity > 18 MΩ·cm) with ultraviolet light and ion exchange treatment. The electrolytic solution pH was ca. 4.0 (20 °C) to 4.3 (80 °C). During the electrodeposition, the solution temperature was kept at a constant value (20, 40, 60 or 80 °C). A gold wire was served as an anode and an Ag/AgCl electrode was used as a reference electrode. Cobalt nanowire arrays were potentio-statically electrodeposited in the AAO nanochannel templates and the time-dependence of the cathode current was monitored to investigate the growth process. During the electrodeposition of nanowires, cathode potentials were kept at −0.75 V, −0.80 V and −0.85 V vs. Ag/AgCl. After the electrodeposition, the AAO nanochannel templates were dissolved in an aqueous solution containing 5 mol L^−1^ NaOH to observe the structure of cobalt nanowire arrays. To avoid etching the surface of the metallic cobalt, the solution temperature was kept at less than 25 °C.

The structure and constituent phases of cobalt nanowire arrays were investigated by field emission scanning electron microscopy (FE-SEM, JSM-7500FA, JEOL Ltd., Tokyo, Japan) and X-ray diffractometer (XRD, Rint-2200, Rigaku Corp, Tokyo, Japan), Cu target, Kα_1_ (λ_1_ = 0.15406 nm) and Kα_2_ (λ_1_ = 0.15444 nm)). Magnetic properties of cobalt nanowire arrays were evaluated by the magnetic hysteresis loops, which were measured using a vibrating sample magnetometer (VSM, TM-VSM1014-CRO, Tamakawa Co., Ltd., Sendai, Japan) at room temperature. During the measurement, the external magnetic field was applied at up to 10 kOe for saturating the magnetization.

## 3. Results

### 3.1. Electrodeposition of Cobalt Nanowire Arrays

**Figure 1:**
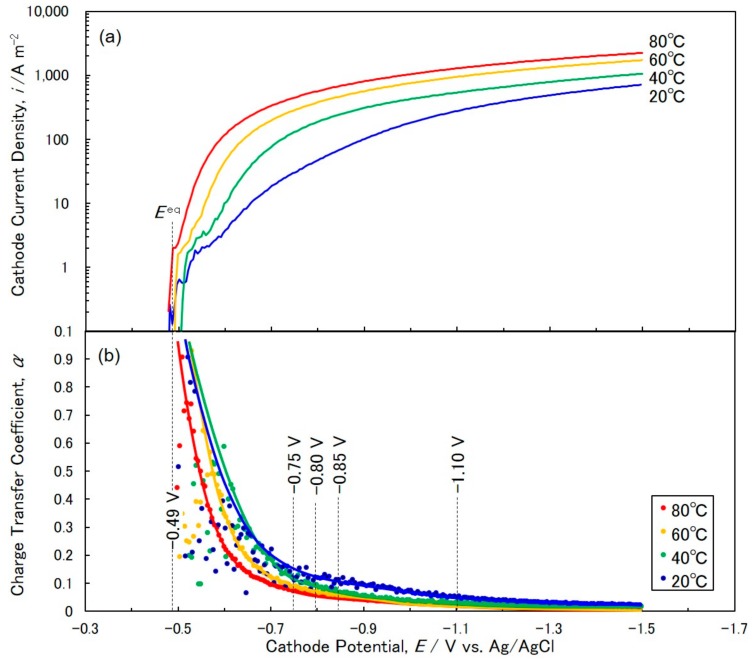
(**a**) Effects of bath temperature on cathode polarization curves and (**b**) the charge transfer coefficient curves for Co electrodeposition from an aqueous solution containing CoSO_4_ and H_3_BO_3_. The bath temperature was kept at 20 °C, 40 °C, 60 °C and 80 °C. The sweep rate was kept at 50 mV s^−1^.

Figure 1a shows the effect of bath temperature on the cathode polarization curves for cobalt electrodeposition on a metallic copper sheet from an aqueous solution containing CoSO_4_ and H_3_BO_3_. The sweep rate was kept at 50 mV s^−1^. In the present experimental condition, the equilibrium potential of Co/Co^2+^ was estimated to be ca. −0.49 V vs. Ag/AgCl (e.g. −0.4878 V at 20 °C, −0.4883 V at 40 °C, −0.4889 V at 60 °C and −0.4895 V at 80 °C) according to Nernst’s equation, as shown in the following Equation (1).

(1) $$E^{eq}=E^{0}+\frac{RT}{nF}ln\frac{\left[M^{n+}\right]}{\left[M^{0}\right]}$$

where *E*^0^ = −0.48 V vs. Ag/AgCl, *R* = 8.3 J K^−1^ mol^−1^, *T* = 293, 313, 333 and 353 K, *n* = 2, *F* = 96,485 C mol^−1^ and [*M*^n+^]/[*M*^0^] = 0.5 (in this study, the activity of metallic cobalt was assumed to be 1). According to Figure 1a, a sharp increase was observed at the cathode potential ca. −0.5 V. This sharp increase of current corresponds to the start of cobalt deposition considering the equilibrium potential of Co/Co^2+^ (−0.49 V vs. Ag/AgCl). By shifting the cathode potential from −0.5 V into the less noble direction, the cathode current density was increased and reached a constant value at ca. −1.5 V (709 A m^−2^ at 20 °C, 1063 A m^−2^ at 40 °C, 1726 A m^−2^ at 60 °C and 2247 A m^−2^ at 80 °C).

Cathodic overpotential, *η*, is expressed by the following Tafel equation:

(2) $$\eta =-\frac{RT}{\alpha nF}ln\left(i^{0}\right)+\frac{RT}{\alpha nF}ln\left(i\right)$$

where *i*^0^ and *i* correspond to the exchange current density and the cathode current density, respectively. By differentiating Equation (2), with respect to the cathode current density, *i*, the charge transfer coefficient, *α*, is expressed by the following equation:

(3) $$\alpha =\frac{RT}{inF}{\left(\frac{d\eta }{di}\right)}^{-1}$$

where d*η*/d*i* is obtained by differentiating the cathode polarization curves (Figure 1a) with respect to the cathode current density, *i*.

Figure 1b shows the effects of bath temperature and cathode potential on the charge transfer coefficient, *α*. Cathode potential ranges nobler than −0.7 V, α fluctuate noticeably due to hydrogen evolution from the reduction of H^+^ ions. In the cathode potential range of −0.7 V to −1.1 V, α decreases by shifting the cathode potential into the less noble direction. Furthermore, in the cathode potential range of −1.1 V to −1.5 V, *α* reaches a constant value due to the diffusion limit of cobalt ions and the decomposition of water solvent [24]. To avoid hydrogen evolution, the optimum cathode potential range for cobalt deposition should be determined, a cathode potential region nobler than that caused the diffusion limit of cobalt ions [24]. Furthermore, in this study, the polarization curves were obtained by employing a metallic copper sheet as a working electrode. However, the narrow channels of AAO templates strongly affected the diffusion of cobalt ions from the bulk electrolyte to the reaction interface. Therefore, the optimum cathode potential for cobalt nanowire deposition should be selected from a range that is less noble than ca. −0.7 V and significantly nobler than −1.1 V to avoid the hydrogen evolution and diffusion limit of cobalt ions. Hence, in this study, a range ca. −0.80 V (−0.75 V, −0.80 V and −0.85 V) was selected as the optimum potential for cobalt nanowires deposition.

**Figure 2:**
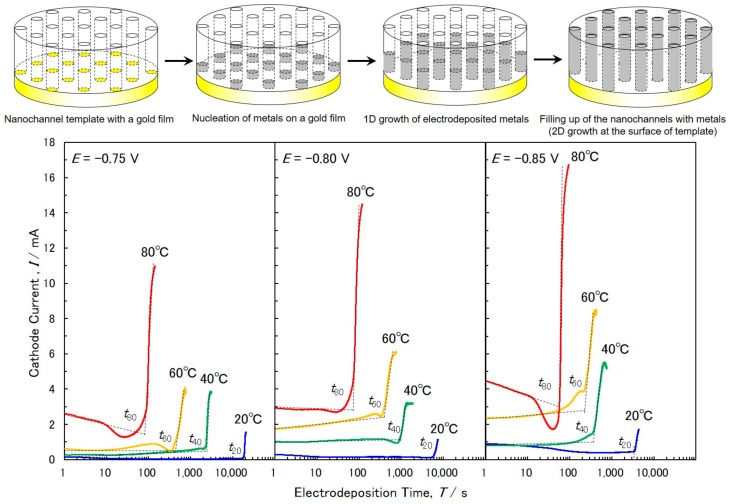
Effects of temperature on the time-dependence of cathode currents during the potentio-static electrodeposition of Co nanowires. The bath temperature was kept at 20, 40, 60 and 80 °C. The cathode potential was kept at −0.75, −0.80 and −0.85 V vs. Ag/AgCl.

Figure 2 shows the effects of temperature on the time-dependence of cathode currents during the electrodeposition of cobalt nanowires in the nanochannels of anodized aluminum oxide templates. At the beginning of the electrodeposition, the cathode current exhibited a stable level due to enough supply of cobalt ions from the bulk of the electrolytic solution to the nanochannels [25]. By increasing the electrodeposition time, a rapid increase in the cathode current was observed under all conditions because of a film-like growth on a surface of a template [26]. After the electrodeposition, this film-like overgrowth was removed by wiping a swab containing nitric acid over the surface. Before removal, the color of the surface was metallic gray. After removal, the color was changed to dark black. Hence, most of the nanowires seemed to reach the surface of template. In this study, a nanochannel template with a thickness of ca. 45 µm was used. The thickness was the same as the lengths of the cobalt nanowires. For instance, at a cathode potential of −0.75 V and a bath temperature of 80 °C, shown in Figure 2, the filling time (time was determined by the point where the extrapolated lines intersected, as shown in Figure 2) was 89.7 s. Hence, the growth rate in these experimental conditions was estimated to be ca. 557 nm s^−1^ (−0.75 V, 80 °C, 50 µm thickness). At a bath temperature of 20 °C, the filling time was ca. 18,000 s which was significantly longer than that observed at 80 °C. Therefore, in these experimental conditions (−0.75 V, 20 °C, 43 µm thickness), the growth rate was estimated to be ca. 2.39 nm s^−1^ which is considerably smaller than that observed at 80 °C. On the other hand, at a cathode potential of −0.85 V, the filling times at a bath temperature of 80 °C and 20 °C were ca. 51.7 s (50 µm thickness) and ca. 3426.8 s (44 µm thickness), respectively. Hence, the growth rates observed at 80 °C and 20 °C were estimated to be ca. 967 nm s^−1^ and ca. 12.8 nm s^−1^, respectively. These growth rates were larger than those observed at −0.75 V. The electrochemical growth rate of metallic nanowires in nanochannels was strongly affected by the diffusion and migration rate of metal ions. The diffusion rate of metal ions increased with an increase in the bath temperature, while the migration rate of metal ions was enhanced by an overpotential for the cathodic reaction. It is well known that the diffusion coefficient of metal ions at different temperatures can generally be predicted by the Arrhenius equation.

**Figure 3:**
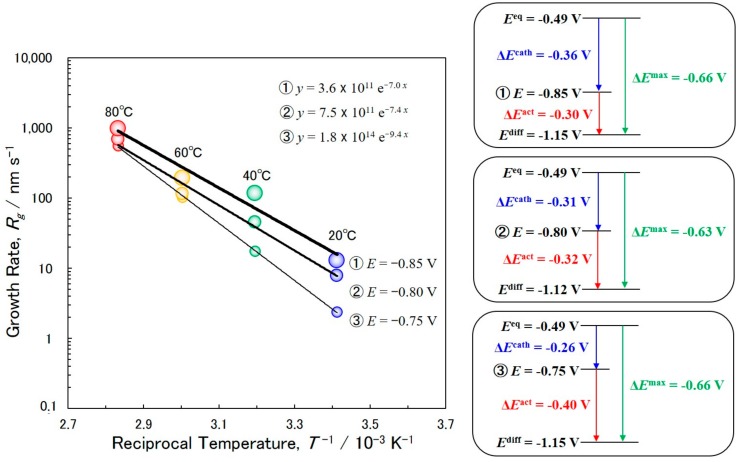
Effects of cathode potential on Arrhenius plots of bath temperature and growth rate of electrodeposited Co nanowires. The schematic figures in the right column represent the relationship between Δ*E*^cath^ and Δ*E*^act^ at different cathode potentials.

Figure 3 shows the effects of cathode potential on the Arrhenius plots between the bath temperature and the growth rate of electrodeposited Co nanowires. The growth rate increases with increased bath temperature and there was an obvious linear relationship between the logarithm of the growth rate (log*R*_g_) and the reciprocal temperature (1/*T*). The slope of the Arrhenius plot corresponds to the activation energy divided by the molar gas constant (−Δ*G*/*R*). The slope varies with cathode potential, according to Figure 3, and the −Δ*G*/*R* at the cathode potentials of −0.75 V, −0.80 V and −0.85 V were determined as −9.4 × 10^3^ K, −7.4 × 10^3^ K and −7.0 × 10^3^ K, respectively. According to the following equation, Δ*G* = −*nF*Δ*E*^act^, the activation overpotentials, Δ*E*^act^, at the cathode potentials of −0.75 V, −0.80 V and −0.85 V are estimated as −0.40 V, −0.32 V and −0.30 V, respectively. According to the equilibrium potential of Co/Co^2+^ (−0.49 V vs. Ag/AgCl), the cathodic overpotential, Δ*E*^cath^, at the cathode potentials of −0.75 V, −0.80 V and −0.85 V are estimated as −0.26 V, −0.31 V and −0.36 V, respectively. Hence, considering the sum of Δ*E*^cath^ and Δ*E*^act^, the maximum activation overpotentials, Δ*E*^max^, at the cathode potentials −0.75 V, −0.80 V and −0.85 V are determined to be ca. −0.66 V, −0.63 V and −0.66 V, respectively. Therefore, at a cathode potential of ca. −1.12 to −1.15 V vs. Ag/AgCl (*E*^diff^), the activation overpotentials, Δ*E*^act^, will be almost zero. According to nucleation theory, number of nuclei can be expressed as follows:

(4) $$N=N_{0}\exp\left(\frac{nF\Delta E^{act}}{RT}\right)$$

where *N* and *N_0_* correspond to the number of nuclei and the maximum number of nuclei, respectively.

According to Equation (4), when Δ*E*^act^ reaches almost zero, *N* will approach a maximum. In this condition, the electrodeposited metallic cobalt will be composed of fine crystals. However, at a less noble cathode potential region, ca. −1.12 to −1.15 V (e.g. −1.5 V), the reduction process of Co^2+^ ions will be controlled by the decomposition process of water solvent.

**Figure 4:**
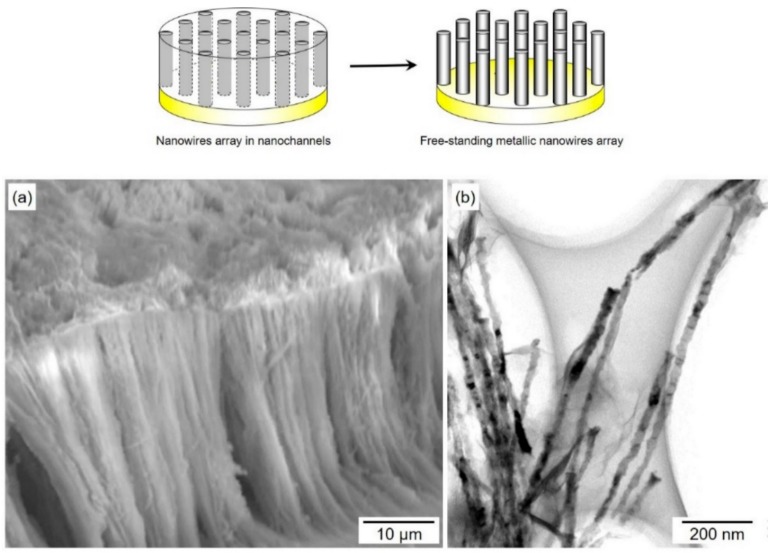
An (**a**) SEM image and (**b**) TEM image of electrodeposited Co nanowire arrays separated by anodized aluminum oxide nanochannel template that was anodized at 20 V.

Figure 4 shows (a) an SEM image and (b) a TEM (Transmission electron microscopy) image of electrochemically grown Co nanowires, which remained after dissolving the anodized aluminum oxide template. The SEM image was observed at a 45° angle against the long axis of nanowires. Each nanowire was free-standing and lay in a parallel direction. The average diameter of the nanowires was estimated by TEM image to be ca. 25 nm. The length of the nanowire, measured by SEM image, was assumed to be ca. 45 µm (32 µm × 1.4). Hence, densely packed Co nanowires with an ultra-large aspect ratio of ca. 1800 were realized in our experimental conditions. Recently, Barriga-Castro et al. revealed that the cylindrical cobalt nanowires, which were electrodeposited at −1.2 V vs. Ag/AgCl into AAO nanochannels, have a pseudo-monocrystalline structure [27]. They investigated the pseudo-monocrystalline structure by SAED (selected area electron diffraction) analysis with HRTEM (high-resolution transmission electron microscopy). According to their report, (111) stacking faults existed along the transverse directions of the cobalt nanowires and these crystalline defects could enhance the crystallization of the fcc phase. As shown in Figure 1, in the cathode potential range of −1.1 V to −1.5 V, the diffusion limit of cobalt ions and the decomposition of water solvent occurred. Therefore, the pseudo-monocrystalline structure could be caused by the diffusion limit of cobalt ions. In this study, the cobalt nanowires were electrodeposited at −0.75 V, −0.80 V and −0.85 V. Hence, the nanowires consisted of a single hcp phase.

### 3.2. Texture and Crystallinity of Co Nanowire Arrays

**Figure 5:**
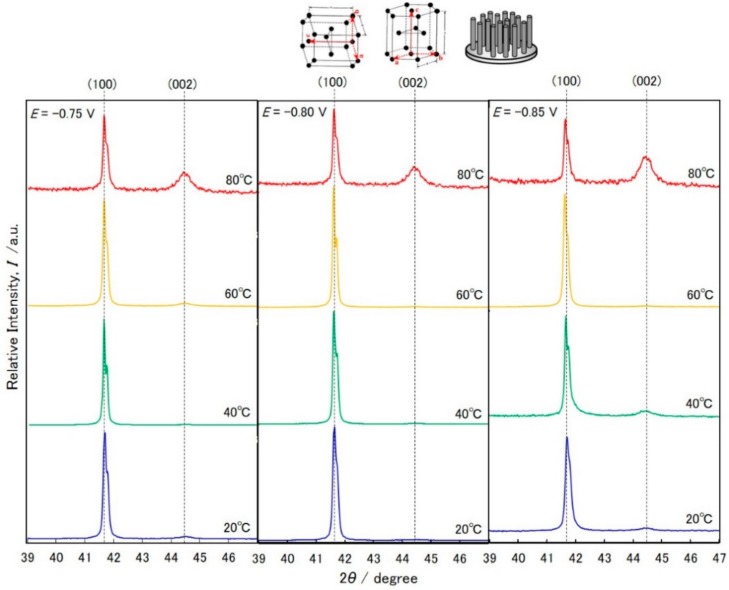
X-ray diffraction patterns of Co nanowire arrays that were electrodeposited at the bath temperatures 20 °C, 40 °C, 60 °C and 80 °C.

Figure 5 shows X-ray diffraction patterns of Co nanowire arrays. As predicted in the previous section, all peaks were assigned to an hcp-Co phase. The peaks corresponding to the fcc-Co phase were not observed. The peak assigned to (100) in hcp-Co (ca. 41.7°) was composed of 2 peaks due to the characteristic X-rays of Kα_1_ (λ_1_ = 0.15406 nm) and Kα_2_ (λ_1_ = 0.15444 nm) from a Cu target. A preferential orientation of (100) in hcp-Co was observed in the all experimental conditions. These results revealed that the c-axis <002> of hcp-Co was not preferentially oriented parallel to the long axis of the Co nanowires. However, the peak assigned to (002) in hcp-Co (ca. 44.4°) was clearly observed in the samples electrodeposited at 80 °C. These results suggest that the c-axis <002> of hcp-Co was partially oriented parallel to the long axis of the Co nanowires obtained at 80 °C. In our previous report, we revealed that the preferential crystal orientation of the c-axis <002> to the long axis of hcp-Co nanowires enhanced the coercivity [22]. Therefore, in this study, the samples obtained at 80 °C showed better magnetic properties compared with those obtained at lower temperatures.

**Figure 6:**
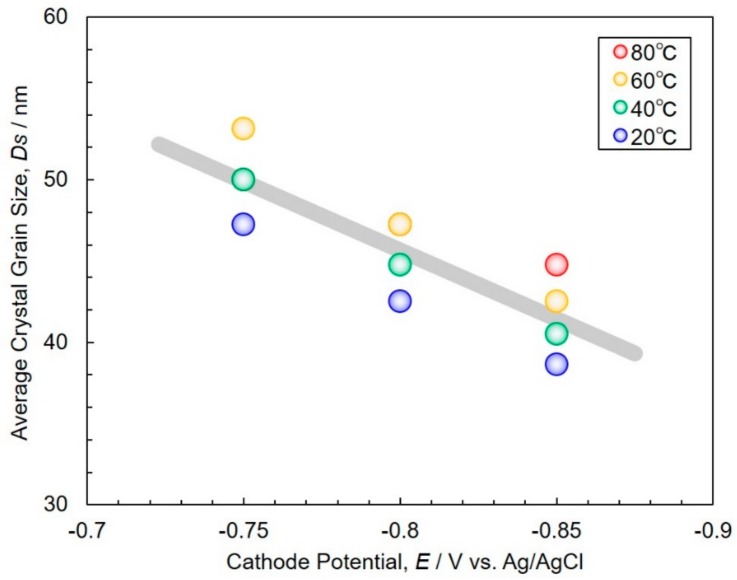
Effects of cathode potential on the average crystal grain size obtained from (100) peaks observed in the X-ray diffraction patterns of Figure 5.

Figure 6 shows the effects of cathode potential on the average crystal grain size, *Ds*, obtained from (100) peaks observed in the X-ray diffraction patterns of Figure 5. *Ds* was determined by the following Scherrer equation.

(5) $$Ds=\frac{K\lambda }{\beta cos\theta }$$

where *K*, *λ*, *β* and *θ* correspond to shape factor (0.9), X-ray wavelength (0.154 nm), hull width at half maximum of (100) peaks and Bragg angle of (100), respectively. *Ds* decreased by increasing the cathodic overpotential, shifting the cathode potential in a less noble direction. It is well known that *Ds* depends on the crystallinity of the samples [28]. *Ds* decreased when the volume of crystal defects, such as dislocations and stacking faults, increased. By increasing the cathodic overpotential, Δ*E*^cath^, during the electrodeposition of Co nanowires, the growth rate of Co nanowires also increased. This is shown in Figure 3. Hence, it is effective for the reduction of crystal defects in Co nanowires to decrease the growth rate of the electrodeposition process.

### 3.3. Magnetization Performance of AAO Nanochannel Films with Co Nanowire Arrays

**Figure 7:**
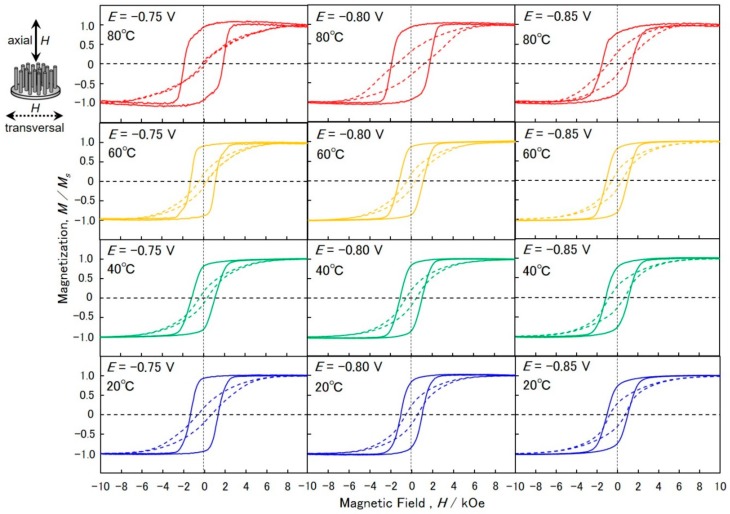
Magnetic hysteresis loops of AAO nanochannel films with Co nanowire arrays that were electrodeposited at bath temperatures of 20, 40, 60 and 80 °C. A magnetic field was applied to axial (solid lines) and transversal (dotted lines) directions of the long axis of nanowires.

Figure 7 shows magnetic hysteresis loops of AAO nanochannel films with Co nanowire arrays. A magnetic field was applied to the axial (solid lines) and transversal (dotted lines) directions of the long axis of nanowires. By increasing the magnetic field in the transversal direction, the magnetization increased gradually and reached saturation at a magnetic field of more than ca. 7 kOe. This result reveals that the films are hardly magnetized in the transversal direction. The coercivity, *H*_c_, and squareness (the ratio of the remnants of the saturated magnetization), *M*_r_/*M*_s_, of films with Co nanowire arrays that were electrodeposited at −0.75 V in a bath temperature of 80 °C are 0.14 kOe and 0.06, respectively. By decreasing the bath temperature to 20 °C, *H*_c_ and *M*_r_/*M*_s_ increased to 0.64 kOe and 0.20, respectively. Furthermore, by shifting the cathode potential in a less noble direction, down to −0.85 V, in a bath temperature of 20 °C, *H*_c_ and *M*_r_/*M*_s_ increased to 0.76 kOe and 0.29, respectively. In this study, the cathode circle area of AAO was ca. 28.26 mm^2^ (circle diameter of ca. 6 mm). The saturation magnetic polarization of a sample was ca. 1 kG. Therefore, if the saturation magnetic polarization of cobalt was 17.9 kG, the space factor of the nanowire arrays was estimated to be ca. 6%.

On the other hand, by increasing the magnetic field in the axial direction, the magnetization increased drastically at a certain magnetic fields and reached saturation at the magnetic field of ca. 3 kOe. This result suggests that the films are easily magnetized in the axial direction, which corresponds to the long-axis of Co nanowire arrays. Comparing the magnetization performance in the axial direction with that observed in the transversal direction, it was found that the axial magnetization behavior of Co nanowire arrays was caused by the shape anisotropy of the nanowires. *H*_c_ and *M*_r_/*M*_s_ of the film with Co nanowire arrays that were electrodeposited at −0.85 V in a bath temperature of 20 °C were 1.02 kOe and 0.72, respectively. By increasing the bath temperature to 80 °C, *H*_c_ and *M*_r_/*M*_s_ increased to 1.44 kOe and 0.74, respectively. Furthermore, by shifting the cathode potential in the noble direction, up to −0.75 V in a bath temperature of 80 °C, *H*_c_ and *M*_r_/*M*_s_ increased to 1.88 kOe and 0.84, respectively.

**Figure 8:**
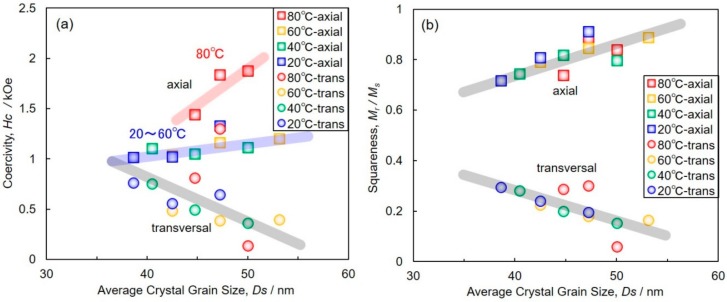
Effects of average crystal grain size on the (**a**) coercivity and (**b**) the squareness of AAO nanochannel films with Co nanowire arrays. A magnetic field was applied to axial and transversal direction of the long axis of nanowires.

Figure 8 shows the effects of average crystal grain size, *Ds*, on (a) the coercivity and (b) the squareness of AAO nanochannel films with Co nanowire arrays. By increasing *Ds*, *H*_c_ and *M*_r_/*M*_s_ in the axial direction increased to 1.88 kOe and 0.91, respectively. On the other hand, when *Ds* increased the *H*_c_ and *M*_r_/*M*_s_ in the transversal direction decreased to 0.14 kOe and 0.06, respectively. As shown in Figure 6, *Ds* increased with a decrease in cathodic overpotential when the cathode potential shifts in the noble direction. This increase in *Ds* corresponds to the reduction of crystal defects in Co nanowires. Therefore, it is suggested that the increase in the *H*_c_ and *M*_r_/*M*_s_ in the axial direction is caused by a reduction of crystal defects in Co nanowires.

In Figure 8a, *H*_c_ in the axial direction of the samples electrodeposited at bath temperatures of 80 °C are greater than those obtained at 20, 40 and 60 °C. According to Figure 5, the peaks assigned to (002) in hcp-Co (ca. 44.4°) are clearly observed in the samples electrodeposited at 80℃. These results suggest that the c-axis <002> of hcp-Co is partially oriented parallel to the long axis of the Co nanowires obtained at 80 °C. Therefore, the increase in *H*_c_ in the axial direction of the samples obtained at 80 °C is also contributed to by the magneto-crystalline anisotropy. This is due to the (002) orientation as well as the reduction of crystal defects.

Fernandez-Roldan et al. investigated the ferromagnetism of Co nanowires and nanotubes with large diameters (*D*: 180 nm) that were electrochemically grown in the AAO templates synthesized by hard anodization at 140 V [29]. They discovered that the *H*_c_ and *M*_r_/*M*_s_ of Co nanowire arrays (*D*: 180 nm, *L*: 6 µm and *L*/*D*: ca. 33) were 0.14 kOe and 0.25, respectively. Zhang et al. studied the growth mechanisms of Co nanowires that were electrochemically grown in the AAO nanochannels using an AC electrodeposition method [30]. They revealed that the *H*_c_ and *M*_r_/*M*_s_ of Co nanowire arrays (*D*: 25 nm, *L*: 2 µm and *L*/*D*: ca. 80) were 1.38 kOe and 0.92, respectively. Khan et al. also examined the ferromagnetism of Co and Co−Fe alloy nanowires that were electrochemically grown in the AAO nanochannels using an AC (14 V, 50 Hz) electrolysis method [31]. They revealed that the *H*_c_ and *M*_r_/*M*_s_ of Co nanowire arrays (*D*: 18 nm, *L*: 3 µm and *L*/*D*: ca. 167) were 1.19 kOe and 0.81, respectively. The maximum *H*_c_ (1.88 kOe) of the Co nanowire arrays obtained in the present study is much greater than that in the reference data reported by other researchers [4,5,8,19,20,21,29,30,31]. This large coercivity in the present study was enhanced by the extremely large aspect ratio of ca. 1800.

## 4. Conclusions

The effects of cathode potential on the Arrhenius plots of the bath temperature and growth rate of electrodeposited Co nanowires was investigated. The activation overpotentials, Δ*E*^act^, at the cathode potentials −0.75 V, −0.80 V and −0.85 V were determined as −0.40 V, −0.32 V and −0.30 V, respectively. Considering the sum of cathodic overpotential, Δ*E*^cath^ and Δ*E*^act^, the maximum activation overpotentials, Δ*E*^max^, were estimated to be ca. −0.63 to −0.66 V. At a cathode potential range less noble than *E*^diff^ (ca. −1.12 to −1.15 V vs. Ag/AgCl), it was predicted that the growth rate of Co nanowires would be controlled by the diffusion process of Co^2+^ ions.

The average diameter, *D*, and length, *L*, of Co nanowires were determined to be ca. 25 nm and ca. 45 µm, respectively. In this study, the aspect ratio, *D*/*L*, of Co nanowires was achieved at up to ca. 1800 and a preferential orientation of (100) in hcp-Co was observed in the all experimental conditions. The average crystal grain size, *Ds*, of (100) decreased with increased Δ*E*^cath^ by a shift of the cathode potential in the less noble direction. It was suggested that the decrease in growth rate of the Co electrodeposition process would be effective for the reduction of crystal defects in Co nanowires.

It was predicted that the perpendicular magnetization behavior of AAO nanochannel films with Co nanowire arrays was caused by the extremely large anisotropy of Co nanowires. By increasing *Ds*, the coercivity, *H*_c_, and squareness, *M*_r_/*M*_s_, of AAO nanochannel films with Co nanowire arrays in the axial direction of magnetic field increased to 1.88 kOe and 0.91, respectively. This study demonstrated the feasibility of improving the magnetic properties of Co nanowire arrays by controlling the degree of Δ*E*^cath^ and crystallinity of Co nanowire arrays.

## Abbreviations

AAO	anodized aluminum oxide
FE-SEM	field emission scanning electron microscopy
hcp	hexagonal close packed structure
VSM	vibrating sample magnetometer
SAED	selected area electron diffraction
HRTEM	high-resolution transmission electron microscopy
